# Association between 5-minute oxygen saturation and neonatal death and intraventricular hemorrhage among extremely preterm infants

**DOI:** 10.1038/s41372-024-02194-w

**Published:** 2024-12-11

**Authors:** Siyuan Jiang, Xin Cui, Anup Katheria, Neil N. Finer, Mihoko V. Bennett, Jochen Profit, Henry C. Lee

**Affiliations:** 1 Division of Neonatology, University of California San Diego, La Jolla, CA USA; 2 Division of Neonatology, Children’s Hospital of Fudan University, Shanghai, China; 3 Division of Neonatology, Stanford University, Stanford, CA USA; 4 California Perinatal Quality Care Collaborative, Stanford, CA USA; 5 Sharp Mary Birch Hospital for Women and Newborns, San Diego, CA USA

**Keywords:** Health services, Respiration

## Abstract

**Objective:**

To assess the relationship between 5-min oxygen saturation (SpO2) and outcomes in extremely preterm infants.

**Study design:**

This cohort study included infants ≤28 weeks’ gestation across nine hospitals from 2020 to 2022. Death and / or severe intraventricular hemorrhage (IVH) were compared between infants with 5-min SpO2 < 80% and 80–100% using Poisson regression models. Receiver Operating Characteristic (ROC) curve and optimal breakpoint analysis were used to estimate the optimal breakpoint of 5-min SpO2 in relation to outcomes.

**Result:**

Of 390 infants, 184 (47.2%) had 5-min SpO2 < 80%. A 5-min SpO2 < 80% was independently associated with increased risks of death and / or severe IVH, early death, and severe IVH. ROC analysis of 5-min SpO2 identified optimal breakpoint at 81–85%, above which no additional benefit in outcomes was observed.

**Conclusion:**

Our findings support the current recommendation of 5-min SpO2 target of ≥80% for extremely preterm infants.

## Introduction

Peripheral oxygen saturation (SpO2) measured by pulse oximetry has become a widely utilized parameter in the delivery room to guide oxygen therapy for extremely preterm infants. Since 2010, clinical guidelines have recommended targeting specific SpO2 levels during resuscitation [1–3]. However, these recommended targets were derived from studies on healthy, term infants following vaginal birth at sea level [4, 5], raising questions about their applicability to extremely preterm infants [6–11]. This uncertainty is further underscored by a survey of 45 clinical practice guidelines, which revealed wide variations in the recommended 5-min SpO2 targets for preterm infants, ranging from 70 to 90% [12]. Given the widespread reliance on these targets, it is crucial to acquire more robust evidence to validate their use.

Most current guidelines recommend a 5-min SpO2 target of 80–85%. A few studies have examined the association between low and high 5-min SpO2 levels and neonatal outcomes, finding that a 5-min SpO2 < 80% is associated with an increased risk of death and intraventricular hemorrhage (IVH) [13–17]. However, these studies often underrepresent infants with the smallest gestational ages (e.g., ≤25 weeks) and did not adjust for initial illness severity. Additionally, most of these studies were conducted over a decade ago, during a period when resuscitation practices have evolved considerably. Furthermore, prior research has typically treated 5-min SpO2 as a dichotomous variable, even though it is a continuous measure ranging from 0 to 100%. The potential existence of an optimal range of 5-min SpO2 that correlates with the lowest risk of adverse outcomes has not been well evaluated.

To address these gaps, our study utilized a contemporary three-year multicenter cohort of extremely preterm infants in California, aiming to: (1) determine the distribution of 5-min SpO2 levels in extremely preterm infants; (2) assess the relationship between 5-min SpO2 < 80% and 80–100% and neonatal outcomes; and (3) explore the continuous relationship between 5-min SpO2 and neonatal outcomes, with the goal of identifying any potential breakpoint associated with the most favorable outcomes.

## Subjects and methods

### Setting

This cohort study utilized data from the Delivery Room Oxygen Quality Initiative of California Perinatal Quality Care Collaborative (CPQCC). The CPQCC, which works with greater than 90% of all NICUs in California, prospectively collects data on infants with a birth weight of 401–1500 g or a gestational age of 22–31 weeks from participating NICUs. The definitions for variables are aligned with those of the Vermont Oxford Network. The Delivery Room Oxygen Quality Initiative is a voluntary research pilot designed to improve the understanding of the ideal initial oxygen concentration for preterm infants. From 2020 to 2022, nine hospitals participated in the initiative, collecting additional variables on delivery room oxygen therapy. Of these hospitals, four were Level IV units, and five were Level III units. Ethical approval for this study was obtained from the Stanford University Institutional Review Board.

### Study population

The study included all infants with gestational age ≤28 weeks who were born at the nine participating units between January 1st, 2020 and December 31st, 2022. Exclusion criteria were delivery room deaths, severe congenital anomalies, and those with missing 5-min SpO2 data.

### Delivery room oxygen therapy and exposure

All infants were resuscitated according to local practice that presumably adhered to the Neonatal Resuscitation Program. The study occurred during a time in which the guidelines recommended that the initial FiO2 be set at 0.21–0.30 for preterm infants. However, the initial respiratory support strategies, SpO2 targets during resuscitation, and FiO2 titration protocols varied by hospital and were not standardized across the initiative. The exposure variable in this study was 5-min SpO2, defined as the average SpO2 at 5 min of life as recorded in the Labor and Delivery record. Time of birth was defined as the time of the complete delivery of the infant.

### Outcomes

The primary outcome was a composite of death before NICU discharge and / or severe IVH. Secondary outcomes included death before NICU discharge, early death ≤7 days of life, death >7 days of life, severe IVH (defined as grade 3 or 4), severe retinopathy of prematurity (ROP, defined as ≥stage 3 or having ROP surgery), and bronchopulmonary dysplasia (BPD, defined as oxygen use at 36 weeks or discharge at 34–35 weeks with supplementary oxygen).

### Statistical analysis

The baseline characteristics were described according to 5-min SpO2 status. Chi-square tests, Fisher’s exact tests, or Wilcoxon-Mann-Whitney tests were performed to compare baseline characteristics between the 5-min SpO2 < 80% group and the 80–100% group. Additionally, the distribution of 5-min SpO2 (median and interquartile range (IQR)) was presented across groups with different baseline factors. Wilcoxon-Mann-Whitney tests (for two group comparisons) or Kruskal–Wallis tests (for comparisons involving more than two groups) were performed to compare the distribution of 5-min SpO2 among infants with different characteristics.

To analyze the association between 5-min SpO2 and the neonatal outcomes of interest, Poisson regression models with robust error variance were fitted to estimate the risk ratios (RRs) and their 95% confidence intervals (95% CIs). We conducted both the crude and multivariable risk adjusted analyses. The risk adjusted analyses included three models. In the primary analysis, model 1 adjusted for gestational age, 1-min Apgar score, and site—confounders chosen for their well-established and significant relationship with both the exposure and outcomes. Model 2 additionally adjusted for 5-min FiO2. Model 3 included a more extended set of confounders, adjusting for gestational age, 1-min Apgar score, site, small for gestational age, congenital malformation, multiple gestation, sex, prenatal care, and delivery by cesarean section.

To explore the nature of the association between 5-min SpO2 and neonatal outcome, 5-min SpO2 was then analyzed as a continuous variable and the association with death and / or severe IVH was explored by fitting two logistic regression models: (1) crude model; and (2) model adjusting for gestational age, 1-min Apgar score, and site. The risk adjusted predicted probabilities obtained from the multivariate logistic regression was used to perform a receiver operating characteristic (ROC) curve analysis to estimate an optimal cutoff value of SpO2 (see detailed method in Supplementary Materials). A two-piecewise regression model was subsequently used to examine the predicted probability function based on the potential cutoff identified from the ROC analysis. In sensitivity analysis, we excluded records with 5-min SpO2 less than 30% and repeated these analyses to investigate the robustness of the results. Analyses were performed using SAS 9.4 (SAS Institute). Statistical significance was set at *p* < 0.05.

## Results

### Study population

A total of 951 infants ≤28 weeks’ gestation were delivered at the participating hospitals from 2020 to 2022. Among these, 72 infants died in the delivery room, 22 had severe congenital anomalies, and 357 were missing data on variables collected for the initiative. Amongst 500 eligible infants with study data, 110 infants with missing data on 5-min SpO2 were excluded from analyses that included 5-min SpO2 (*n* = 390) (Supplementary Fig. 1: flow chart of the population). The baseline characteristics of infants with and without 5-min SpO2 data were similar (Supplementary Table 1), with the exception that infants with available 5-min SpO2 had a lower rate of delayed cord clamping, higher 5-min FiO2 and lower 5-min Apgar score.

Among the cohort of 390 infants, the median gestational age was 26.0 (IQR 24.0-27.0) weeks, and the median birth weight was 879.9 (IQR 675.0-1055.0) grams.

### Distribution of 5-min SpO2 and associated factors

**Fig. 1:**
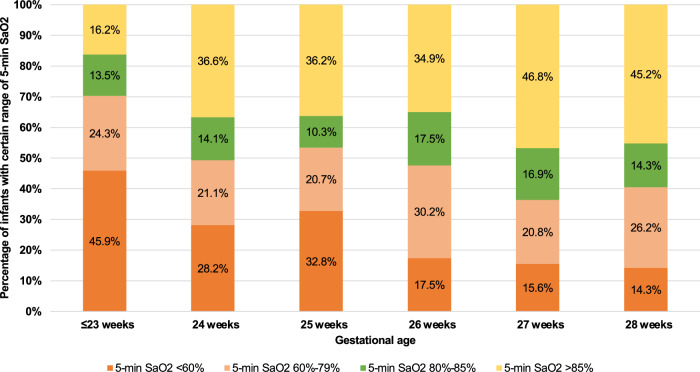
Distribution of 5-min SpO2 by gestational age among extremely preterm infants.

The median SpO2 at 5 min of life across all infants was 80% (IQR 60-90%). Overall, 23.3%, 23.8%, 14.6% and 38.2% infants had 5-min SpO2 < 60%, 60–79%, 80–85%, and >85%, respectively. The median 5-min SpO2 increased with gestational age (Table 1). Specifically, 70.3% of infants born at ≤23 weeks, 50.0% of those born at 24-26 weeks, and 38.5% of those at 27–28 weeks had a 5-min SpO2 of <80% (Fig. 1). The 5-min SpO2 also varied across the participating sites, ranging from a median of 61.5% (IQR 36.0–82.5) to 90.0% (IQR 90.0–90.0) among different hospitals (Table 1, Supplementary Fig. 2). The 5-min SpO2 was not significantly associated with other infant characteristics, except 1-min Apgar score (Table 1).

**Table 1:** Factors associated with 5-min SpO2.

	*N*	Median (interquartile range)	*P* value
Overall	390	80.0 (60.0–90.0)	
Gestational age			
≤23 weeks	37	61.0 (37.0-83.0)	**0.002**
24 weeks	71	80.0 (53.0–90.0)	
25 weeks	58	75.0 (50.0–90.0)	
26 weeks	63	80.0 (65.0–90.0)	
27 weeks	77	85.0 (67.0-91.0)	
28 weeks	84	85.0 (71.5-91.5)	
Small for gestational age			
No	353	80.0 (60.0–90.0)	0.623
Yes	37	84.0 (62.0–90.0)	
Sex			
Male	209	79.0 (60.0–90.0)	0.367
Female	181	82.0 (65.0–90.0)	
Multiple births			
Singleton	318	80.0 (60.0–90.0)	0.960
First twin	34	80.0 (67.0–90.0)	
Second or third twin	38	79.5 (60.0-91.0)	
Non-reassuring fetal status			
No	271	80.0 (65.0–90.0)	0.475
Yes	119	78.0 (57.0–90.0)	
Antenatal steroids			
No	21	82.0 (46.0–84.0)	0.251
Yes	332	80.0 (65.0–91.0)	
Antenatal MgSO_4_			
No	65	82.0 (54.0–90.0)	0.793
Yes	325	80.0 (60.0–90.0)	
Mode of delivery			
Vaginal delivery	110	78.0 (60.0–90.0)	0.276
Cesarean section	280	80.0 (61.0–91.0)	
Delayed cord clamping			
No	190	70.1 (52.0–90.0)	0.094
30–60 s	160	84.0 (68.0–90.0)	
61–120 s	40	81.0 (61.5–92.0)	
Cord milking			
No	366	80.0 (60.0–90.0)	0.667
Yes	24	75.5 (57.0–90.5)	
1-min Apgar score			
0–3	150	70.0 (49.0–85.0)	**<.001**
4–6	150	82.5 (65.0–91.0)	
7–10	89	87.0 (79.0–93.0)	
Site			
A	29	80.0 (50.0–90.0)	**0.021**
B	116	73.5 (56.0–87.0)	
C	73	80.0 (68.0–90.0)	
D	23	85.0 (80.0–90.0)	
E	76	82.5 (60.0–93.5)	
F	17	90.0 (90.0–90.0)	
G	^a^	61.5 (36.0–82.5)	
H	39	84.0 (56.0–90.0)	
I	^a^	75.0 (73.0–89.0)	

The bold values indicate *P* < 0.05, signifying statistical significance.

^a^Cell counts <12 were suppressed in order to protect subject privacy.

### High (80–100%) and low (<80%) 5-min SpO2 and neonatal outcomes

Overall, 47.2% (184/390) infants had a 5-min SpO2 < 80%, while 52.8% (206/390) had a 5-min SpO2 of 80–100%. Compared to infants with a higher 5-min SpO2, those with a low SpO2 (<80%) had significantly lower gestational age and birth weight (Table 2). During delivery room resuscitation, infants with low 5-min SpO2 also had significantly lower 1-min and 5-min Apgar scores, lower rates of nasal continuous positive airway pressure, higher rates of endotracheal tube ventilation, and higher 5-min FiO2 (Table 2).

**Table 2:** Baseline characteristics by 5-min SpO2.

	5-min SpO2 < 80%	5-min SpO2 80–100%	*P* value
**Number of infants**	184	206	
**Infant characteristics**			
Gestational age, median (interquartile range), weeks	25.5 (24.0-27.0)	26.0 (25.0-27.0)	**0.002**
≤25 weeks, n (%)	92 (50.0)	74 (35.9)	**0.022**
26 weeks, n (%)	30 (16.3)	33 (16.0)	
27 weeks, n (%)	28 (15.2)	49 (23.8)	
28 weeks, n (%)	34 (18.5)	50 (24.3)	
Birth weight, median (interquartile range), grams	815.5 (643.0-1000.0)	879.0 (705.0-1095.0)	**0.005**
Small for gestational age, n (%)	16 (8.7)	21 (10.2)	0.614
Male, n (%)	106 (57.6)	103 (50.0)	0.133
Multiple births, n (%)	35 (19.0)	37 (18.0)	0.788
**Maternal characteristics**			
Prenatal care, n (%)	177 (96.2)	197 (95.6)	0.779
Maternal hypertensive disorders, n (%)	43 (23.4)	56 (27.2)	0.388
Maternal diabetes, n (%)	23 (12.5)	30 (14.6)	0.553
Non-reassuring fetal status, n (%)	61 (33.2)	58 (28.2)	0.285
Antenatal steroids, n (%)^a^	150 (94.9)	182 (93.3)	0.527
Antenatal MgSO_4_, n (%)	157 (85.3)	168 (81.6)	0.318
Cesarean delivery, n (%)	128 (69.6)	152 (73.8)	0.355
**Delivery room resuscitation**			
Delayed cord clamping, n (%)	86 (46.7)	114 (55.3)	0.090
No, n (%)	98 (53.3)	92 (44.7)	0.194
30–60 s, n (%)	67 (36.4)	93 (45.1)	
61–120 s, n (%)	19 (10.3)	21 (10.2)	
Cord milking, n (%)	14 (7.6)	^b^	0.259
Nasal continuous positive airway pressure, n (%)	107 (58.2)	168 (81.6)	**<0.001**
PPV via mask and / or noninvasive ventilation, n (%)	158 (85.9)	148 (71.8)	**0.001**
Endotracheal tube ventilation, n (%)	109 (59.2)	63 (30.6)	**<0.001**
Chest compression and / or epinephrine, n (%)	^b^	^b^	0.262
5-min FiO2, median (interquartile range)	80.0 (40.0-100.0)	40.0 (30.0-60.0)	**<0.001**
0.21-0.30, n (%)	31 (16.8)	71 (34.5)	**<0.001**
0.31-0.60, n (%)	50 (27.2)	84 (40.8)	
0.61-1.0, n (%)	102 (55.4)	51 (24.8)	
1-min Apgar score, median (interquartile range)	3.0 (2.0–5.0)	5.0 (3.0–7.0)	**<0.001**
0–3, n (%)	94 (51.1)	56 (27.2)	**<0.001**
4–6, n (%)	64 (34.8)	86 (41.7)	
7–10, n (%)	25 (13.6)	64 (31.1)	
5-min Apgar score, median (interquartile range)	6.0 (4.0–8.0)	8.0 (7.0–8.0)	**<0.001**
0–3, n (%)	33 (17.9)	^b^	**<0.001**
4–6, n (%)	62 (33.7)	^b^	
7–10, n (%)	89 (48.4)	165 (80.1)	

The bold values indicate *P* < 0.05, signifying statistical significance.

^a^Data on antenatal steroids missing for *n* = 37 infants; % calculated based on non-missing value.

^b^Cell counts <12 were suppressed in order to protect subject privacy. Data may not add up to 100% due to missing data.

A total of 23.4% (43/184) of infants in the low 5-min SpO2 group and 11.7% (24/206) in the high 5-min SpO2 group developed the composite outcome of death and / or severe IVH (crude RR 2.01, 95% CI 1.27, 3.18) (Table 3). Infants with 5-min SpO2 < 80% also had higher risks of overall death, early death ≤7 days of life, and severe IVH compared to those in the high 5-min SpO2 group. After adjusting for gestational age, 1-min Apgar score and site, a low 5-min SpO2 was independently associated with increased risks of the composite outcome (adjusted RR 1.65, 95% CI 1.03–2.63), early death (adjusted RR 3.08, 95% CI 1.02–9.32) and severe IVH (adjusted RR 2.32, 1.07–4.99). These associations remained significant after further adjustment for 5-min FiO2 in Model 2. After adjusting for additional perinatal characteristics in Model 3, the direction of the associations remained consistent, although they became marginally non-significant for the composite outcome and severe IVH.

**Table 3:** Association between 5-min SpO2 and neonatal outcomes.

	5-min SpO2 < 80% *n* = 184	5-min SpO2 80–100% *n* = 206	Crude RR (95% CI)	Model 1 adjusted RR^a^ (95% CI)	Model 2 adjusted RR^b^ (95% CI)	Model 3 adjusted RR^c^ (95% CI)
Death and / or severe intraventricular hemorrhage, n (%)	43 (23.4)	24 (11.7)	**2.01 (1.27, 3.18)**	**1.65 (1.03, 2.63)**	**1.65 (1.03, 2.63)**	1.48 (0.92, 2.38)
Death, n (%)	28 (15.2)	15 (7.3)	**2.09 (1.15, 3.80)**	1.60 (0.84, 3.06)	1.61 (0.85, 3.04)	1.46 (0.76, 2.79)
Death ≤7 days of life, n (%)	^d^	^d^	**4.03 (1.52, 10.67)**	**3.08 (1.02, 9.32)**	**3.05 (1.07, 8.71)**	**2.86 (1.11, 7.38)**
Death >7 days of life, n (%)	^d^	^d^	1.12 (0.48, 2.64)	0.81 (0.31, 2.11)	0.83 (0.33, 2.11)	0.83 (0.30, 2.33)
Severe intraventricular hemorrhage, n (%)	23 (12.5)	12 (5.8)	**2.26 (1.16, 4.41)**	**2.32 (1.07, 4.99)**	**2.10 (1.00, 4.43)**	2.07 (0.98, 4.37)
Severe retinopathy of prematurity, n (%)	17 (9.2)	13 (6.3)	1.66 (0.83, 3.30)	1.12 (0.54, 2.31)	0.97 (0.46, 2.02)	1.12 (0.53, 2.35)
Bronchopulmonary dysplasia, n (%)	84 (45.7)	90 (43.7)	1.21 (0.98, 1.48)	1.07 (0.87, 1.32)	1.02 (0.83, 1.25)	1.06 (0.86, 1.30)

*RR* risk ratio, *CI* confidence interval.

^a^Adjusted for gestational age, 1-min Apgar score, and site.

^b^Adjusted for gestational age, 1-min Apgar score, site and 5-min FiO2.

^c^Adjusted for gestational age, 1-min Apgar score, site, small for gestational age, congenital malformation, multiple gestation, gender, prenatal care, and delivery by cesarean section.

^d^Cell counts <12 were suppressed in order to protect subject privacy.

### 5-min SpO2 as a continuous variable and its association with neonatal outcomes

**Fig. 2:**
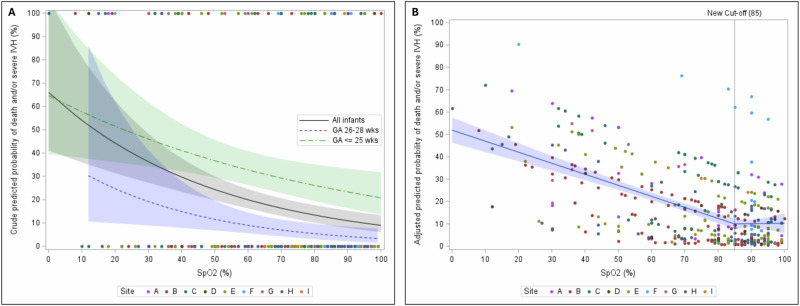
Association of predicted probability of death and / or severe intraventricular hemorrhage and 5-min SpO2. **A** Crude predicted probability of death and / or severe IVH and 5-min SpO2. Scatter plot: Binary (1=yeas, 0=no) death and / or severe IVH by 5-min SpO2; Fitted lines with 95% confidence intervals. **B** Adjusted predicted probability of death and / or severe IVH and 5-min SpO2: An optimal breakpoint was identified at a 5-min SpO2 of 85%. Below this 85% threshold, the adjusted probability of death and / or severe IVH significantly decreased with increasing 5-min SpO2 (slope -5.1%, 95% CI (–6.1%, –4.2%), *P* value < 0.001); while above 85%, the adjusted probability of death and / or severe IVH was not significantly associated with 5-min SpO2 (slope 0.7%, 95% CI (–1.7%, 3.1%), *P* value = 0.572). Scatter plot: Predicted probability of death and / or severe IVH by 5-min SpO2; Fitted lines with 95% confidence intervals.

The estimated crude probability of death and / or severe IVH as a function of 5-min SpO2, treated as a continuous variable, is presented in Fig. 2. Overall, the risk of death and / or severe IVH decreased as 5-min SpO2 increased. When stratified by gestational age, the risk curves for infants <26 weeks and those 26–28 weeks were nearly parallel (Fig. 2A).

The ROC analysis identified 85% as the optimal breakpoint using the risk adjusted model (detailed results in Supplementary Materials). When 5-min SpO2 was lower than or equal to 85%, each 10% increase in 5-min SpO2 corresponded to a 5.1% reduction in the predicted probability of death and / or severe IVH (slope –5.1%, 95% CI (–6.1%, –4.2%), *P* value < 0.001). While above 85%, the predicted probability of death and / or severe IVH did not change significantly (slope 0.7%, 95% CI (–1.7%, 3.1%), *P* value = 0.572) (Fig. 2B). In the sensitivity analysis that excluded records with 5-min SpO2 less than 30%, a similar optimal breakpoint was found at 81% (Supplementary Fig. 3 and Supplementary Fig. 4B).

## Discussion

In this multicenter cohort study of extremely preterm infants, nearly half did not achieve the recommended 5-min SpO2 target of 80%. We confirmed that a 5-min SpO2 < 80% is independently associated with an increased risk of adverse neonatal outcomes, including early death and severe IVH. The analysis of the non-linear relationship identified a breakpoint at 81–85% for 5-min SpO2, with continuous improvement in outcomes observed up to this threshold.

In our cohort of extremely preterm infants, 47% did not achieve the recommended SpO2 target at 5 min after birth, aligning with previous studies that reported 46–48% of very preterm infants with 5-min SpO2 < 80% [13, 16, 18]. This indicates that despite nearly two decades of efforts following the initial guideline recommendation on SpO2 targets, a substantial proportion of extremely preterm infants still fail to meet the recommended target. This phenomenon raises several questions that warrant further investigation. First, given the large number of infants who did not reach the target, the appropriateness of the SpO2 target for this specific gestational age group requires further validation. It is also noteworthy that much fewer infants were within the recommended range of 5-min SpO2 at 80–85% in both our study and previous reports [19]. Considering the lack of evidence, the implications and validity of the upper limit of 5-min SpO2 target also deserve careful scrutiny. Second, efforts should be made to increase the proportion of extremely preterm infants who achieve the 80% goal for 5-min SpO2, especially before more evidence emerges to support alternative targets. All participating hospitals in our study would have adopted the low initial oxygen strategy recommended by current guidelines. However, a recent large meta-analysis found reduced risk of mortality in infants resuscitated with high initial oxygen compared to those with low initial oxygen, challenging current practice [19]. Additionally, we also found 17% and 27% infants with 5-min SpO2 < 80% received only 0.21–0.30 and 0.31-0.60 FiO2 at 5 min of life, suggesting conservative FiO2 titration and potentially insufficient oxygen supply for initially hypoxic infants [12, 13, 17, 20]. The delay in obtaining accurate SpO2 readings—which may be partly due to delayed cord clamping, and the time required for an oximeter to function—means that the first indication of a low SpO2 may not appear until 2 or 3 min after birth. This narrow window leaves little time for corrective action and could contribute to the low rate of successful achievement of SpO2 target, indicating a potential need for a change in resuscitation practices. Furthermore, though our study did not show a significant association between cord management and 5-min SpO2, studies have suggested that delayed cord clamping was associated with lower SpO2 levels [21]. The optimal oxygen therapy that incorporates current cord management practices also requires further investigation.

Our study further confirmed that 5-min SpO2 < 80% is associated with adverse outcomes, particularly early death ≤ 7 days and severe IVH [13, 14, 16, 17, 22]. Compared to previous research, our study enrolled more infants ≤25 weeks and adjusted for initial illness severity using 1-min Apgar score. As a result, our findings may provide additional insights into the generalizability of this association to the smallest infants, as well as the independent effect of SpO2 irrespective to illness severity. Moreover, in our second multivariable model, we further adjusted for 5-min FiO2 to better illustrate the association of SpO2 and outcomes independent of oxygen supply. The persistent significant association may suggest that SpO2 targets are important regardless of the amount of oxygen that is administered. However, caution is warranted in interpreting these results. Residual confounding related to initial illness severity may still be present. More importantly, as with all previous observational studies, we cannot establish a causal relationship between 5-min SpO2 and outcomes. However, in current clinical practice, the use of SpO2 as a target for oxygen therapy presumes a casual effect, wherein an increase in 5-min SpO2 is expected to lead to improved outcomes. Given the widespread adoption of SpO2-targeted therapy without definitive evidence, there is an urgent need for interventional studies to evaluate the direct impact of different SpO2 targets on neonatal outcomes.

Unlike previous studies, we examined the association between 5-min SpO2 as a continuous variable and the risk of adverse outcomes. The current recommendation of 5-min SpO2 goal is based on the “normal” range observed in healthy, term infants. However, the optimal target may be one that leads to the best outcomes rather than simply mirroring “normative” data. To explore this, we illustrated the probability of adverse outcomes in relation with 5-min SpO2 ranging from 0 to 100%, with the aim of identifying a range of SpO2 associated with the lowest risk. Initially, we hypothesized a U-shaped relationship due to concerns about both toxic hypoxia and hyperoxia [9, 10]. However, our findings revealed that the crude risk of adverse outcomes continuously decreased as 5-min SpO2 increased. The absence of increased risk at the higher end of SpO2 spectrum could be attributed to the fact that more initially healthier infants were among those with the highest 5-min SpO2. Additionally, given the limited sample size, our study was underpowered to compare outcomes between infants with a 5-min SpO2 > 85% and those in the 80–85% range. With the most recent meta-analysis showing a preference for high FiO_2_ in the delivery room, future studies are needed to further explore the effects of higher oxygen saturations [18].

In the analysis to further explore whether the independent benefit of increasing SpO2 might diminish or reverse beyond a certain point, we found that a breakpoint at 81–85% for the 5-min SpO2 does exist. This breakpoint aligns closely with the current recommendation on a 5-min targets of 80–85%. This finding lends additional support to the recommendation of targeting a relatively high 5-min SpO2, as each percentage increase of 5-min SpO2 up to the breakpoint was associated with a reduced risk of death and / or IVH. On the other hand, the absence of additional benefit when 5-min SpO2 exceeds 85% suggests that overshooting this target may not be necessary. While equally important, it also reassures that mortality and severe IVH at least do not increase with higher SpO2, challenging strategies that aim to avoid hyperoxia at potential cost of increased low SpO2 incidence [19]. However, our study should be viewed as hypothesis-generating, as several key questions remain to be addressed by well-designed interventional studies with sufficient sample size. The effects of a higher SpO2 target also warrant further larger-scale investigation. Additionally, we suspect that SpO2 targets may need to be individualized for different groups of infants. The optimal targets may vary based on gestational age, illness severity at birth, or the type of respiratory support required.

There are limitations to this study. Most importantly, the observational nature limited our ability to establish a causal relationship between 5-min SpO2 and neonatal outcomes. Second, 22% of eligible extremely preterm infants did not have 5-min SpO2 data. Though the missing data group had very similar baseline characteristics to the analyzed group, they had slightly higher Apgar scores and lower 5-min FiO2 requirements. As a result, we may have slightly overestimated the failure rate of achieving the 5-min SpO2 target. Third, delivery room deaths were not included in the study, which may have led to an underestimation of the impact of lower SpO2 on adverse outcomes. Fourth, the measurement and recording of 5-min SpO2 were not standardized across participating hospitals, potentially introducing measurement bias.

Our study supports the current recommendation of a 5-min SpO2 target of ≥80% for extremely preterm infants, as failing to reach this threshold is associated with significantly higher risks of early death and severe IVH. There may be no significant additional benefit beyond certain SpO2 level, requiring further confirmation. These findings reinforce the importance of optimizing early oxygen management while also highlighting the need for further interventional research to refine and individualize SpO2 targets for this vulnerable population.

## Supplementary information

Supplemental Material

## Data Availability

The data that support the findings of this study are available from CPQCC but restrictions apply to the availability of these data, which were used under license for the current study, and so are not publicly available. Data are however available from the authors upon reasonable request and with permission of CPQCC.
