# Supplementary material for: Association between 5-minute oxygen saturation and neonatal death and intraventricular hemorrhage among extremely preterm infants
Source: J Perinatol. 2024 Dec 11;45(8):1145–51. doi: 10.1038/s41372-024-02194-w (PMC12367537; doi:10.1038/s41372-024-02194-w)
Supplement: Supplementary file 1 — Supplemental Material [file 41372_2024_2194_MOESM1_ESM.docx]

**Supplemental Materials**

1. **Statistical method on optimal cutoff value selection for SPO2**
2. **Detailed results on optimal cutoff value selection for SPO2**
3. **Supplemental Table 1. Comparison of infants with and without 5-minute SaO2 data**
4. **Supplemental Figure 1. Study Population**
5. **Supplemental Figure 2. Distribution of 5-min SpO2 among extremely preterm infants in participating sites**
6. **Supplemental Figure 3. Adjusted predicted probability of death and / or severe IVH and 5-minute SpO2 by excluding records with 5-minute SpO2 less than 30%**
7. **Supplementary Figure 4. ROC curve and optimal breakpoint identified from the ROC analysis**

**Statistical method on optimal cutoff value selection for SpO2**

To explore the nature of the association between 5-minute SpO2 and neonatal outcome, 5-minute SpO2 was then analyzed as a continuous variable and the association with death and / or severe IVH was explored by fitting separate logistic regression models: 1) crude model; and 2) model adjusting for gestational age, 1-min Apgar score, and site. The risk adjusted predicted probabilities obtained from the multivariable logistic regression was used to perform a receiver operating characteristic (ROC) curve analysis to estimate an optimal cutoff value of SpO2^[1]^. Different cutoff points were first chosen based on a simultaneous assessment of sensitivity and specificity with balancing of these two measures. Setting a high SpO2 cutoff value will decrease the number of false negatives but increase false positives. Some of the popular methods include criteria based on: 1) correct classification rate; 2) the distance to the (0, 1) point on the ROC curve, 3) the Youden index, defined as sensitivity + specificity – 1, which is the height above the uninformative diagonal on the ROC curve; and 4) the absolute difference between sensitivity and specificity. Based on the potential cutoff candidates identified from the ROC analysis, a two-piecewise regression model was subsequently used to examine the predicted probability function under different model assumptions: 1) a natural cubic model with quadratic relationship on the first segment and linear relationship on the second segment; and 2) a linear model with linear relationship on both segments. The final breakpoint was determined by examining the fitted slopes between 5-minute SpO2 and predicted probability of death and / or severe intraventricular hemorrhage (IVH) on both sides of the breakpoint candidates. The estimated slopes quantify an abrupt change in the predicted probabilities, and the breakpoint (knot) can be interpreted as a critical value below which infant outcome is likely to be affected, and can be important in clinical decision making. In sensitivity analysis, we excluded records with 5-minute SpO2 less than 30% and repeated these analyses to investigate the robustness of the results. The SAS macro ROCPLOT was used to perform the ROC analysis^[2]^, and GLIMMIX procedure with Laplace estimation and spline effect was used to fit the two-piecewise regression models.

**Detailed results on optimal cutoff value selection for SpO2**

In the ROC analysis using the risk adjusted model, the area under the curve 0.82 was achieved, and multiple 5-minute SpO2 breakpoint candidates were identified by various criteria, specifically: 1) The breakpoint at 5-minute SpO2 90% gave the highest correct classification rate (0.835) and minimized the absolute difference between sensitivity and specificity (0.005); 2) The breakpoint at 5-minute SpO2 91% minimized the distance to the "perfect" point at the upper-left of the ROC plot (0.382); and 3) The breakpoint at 5-minute SpO2 59% and 85% maximized the Youden index (both 0.535). It is not uncommon to find multiple cutoff points, and determination of the final cutoff choice is context-dependent while the ROC analysis identifies them from statistical quantitative-based measures^[3]^. In the evaluation of the cutoff candidates using the spline models, the natural cubic model and the linear model led to similar of fit statistics based on Pearson Chi-square divided by degrees of freedom (both 0.93; close to 1 suggesting a good fit), therefore the simpler linear spline piecewise model was chosen with using the identified cutoff point as pre-specified knot to estimate the slopes between 5-minute SpO2 and the adjusted predicted probability of death and / or severe IVH. At the breakpoint of 5-minute SpO2 59%, for every 10% increase of 5-minute SpO2, the predicted probability of death and / or severe IVH decreased significantly on both sides of the breakpoint (slope -6.8%, 95% confidence interval (CI) (-9.5%, -4.1%), P-value < 0.001 when 5-minute SpO2 < 59%; slope -2.3%, 95% CI (-2.9%, -1.8%), P-value < 0.001 for 5-minute SpO2 > 59%), indicating that the likelihood of death and / or severe IVH continuously decreased as 5-minute SpO2 increased although the rate of the decrease has changed. At the breakpoint of 5-minute SpO2 85%, for every 10% increase of 5-minute SpO2, the predicted probability of death and / or severe IVH decreased when 5-minute SpO2 was lower than or equal to 85% (slope -5.1%, 95% CI (-6.1%, -4.2%), P-value < 0.001); Above 85%, the predicted probability of death and / or severe IVH did not change (slope 0.7%, 95% CI (-1.7%, 3.1%), P-value = 0.572) (Figure 2 Panel B). Similar results were found for breakpoint candidate of 5-minute SpO2 at 90% and 91%, where the slope was significantly lower than zero on the left side of the breakpoint but not different from zero on the right side of the breakpoint. These results indicated that the likelihood of developing death and / or severe IVH decreased when 5-minute SpO2 increased from 0 to 85% but did not change after 85%. Based on the estimates of the slopes for each breakpoint candidate, we identified that 85% was the optimal breakpoint in our analysis. This breakpoint is the lowest cutoff point where there is no more decrease in the predicted probabilities beyond this point (estimated probability 9.7%) and maximizes both sensitivity and specificity based on Youden Index (97.0% sensitivity and 56.2% specificity) (Supplemental Figure 4 Panel A). The high sensitivity and lower specificity indicate that the model will identify the outcome in most infants who have it, but it may also have high likelihood to falsely identify the outcome in a number of infants. In sensitivity analysis, by excluding records with 5-minute SpO2 less than 30%, similar optimal breakpoint was found at 81%. At the breakpoint of 5-minute SpO2 81%, for every 10% increase of 5-minute SpO2, the predicted probability of death and / or severe IVH decreased when 5-minute SpO2 was lower than or equal to 81% (slope -4.6%, 95% CI (-6.0%, -3.3%), P-value < 0.001); Above 81%, the predicted probability of death and / or severe IVH did not change (slope -0.8%, 95% CI (-2.6%, 1.0%), P-value = 0.369) (Supplemental Figure 3 and Supplemental Figure 4 Panel B).

**References**

1. Unal I. Defining an optimal cut-point value in roc analysis: An alternative approach. *Comput Math Methods Med* 2017, **2017:** 3762651.

2. Plot roc curve with cutpoint labeling and optimal cutpoint analysis. [accessed 2024 Oct, 20] Available from: <https://support.sas.com/kb/25/018.html>

3. Perkins NJ, Schisterman EF. The inconsistency of "optimal" cutpoints obtained using two criteria based on the receiver operating characteristic curve. *Am J Epidemiol* 2006, **163**(7)**:** 670-675.

**Supplemental Table 1. Comparison of infants with and without 5-minute SaO2 data**

|  | **5-minute SaO2  available** | **5-minute SaO2  missing** | **P-value ^1^** |
| --- | --- | --- | --- |
| **Number of infants** | 390 | 110 |  |
|  |  |  |  |
| **Infant characteristics** |  |  |  |
| Gestational age, median (interquartile range), weeks | 26.0 (24.0-27.0) | 26.0 (25.0-27.0) | 0.640 |
| ≤25 weeks, n (%) | 166 (42.6) | 41 (37.3) | 0.211 |
| 26 weeks, n (%) | 63 (16.2) | 17 (15.5) |  |
| 27 weeks, n (%) | 77 (19.7) | 32 (29.1) |  |
| 28 weeks, n (%) | 84 (21.5) | 20 (18.2) |  |
| Birth weight, median (interquartile range), grams | 879.9 (675.0-1055.0) | 895.0 (695.0-1050.0) | 0.986 |
| Small for gestational age, n (%) | 37 (9.5) | 12 (10.9) | 0.658 |
| Male, n (%) | 209 (53.6)) | 54 (49.1) | 0.404 |
| Multiple births, n (%) | 72 (18.5) | 28 (25.5) | 0.105 |
|  |  |  |  |
| **Maternal Characteristics** |  |  |  |
| Prenatal care, n (%) | 374 (95.9) | 105 (95.5) | 0.838 |
| Maternal hypertensive disorders, n (%) | 99 (25.4) | 24 (21.8) | 0.443 |
| Maternal diabetes, n (%) | 53 (13.6) | 17 (15.5) | 0.619 |
| Non-reassuring fetal status, n (%) | 119 (30.5) | 29 (26.4) | 0.340 |
| Antenatal steroids, n (%) | 332 (94.1) | 95 (96.9) | 0.260 |
| Antenatal MgSO_4_, n (%) | 325 (83.3) | 91 (82.7) | 0.881 |
| Cesarean delivery, n (%) | 280 (71.8) | 81 (73.6) | 0.703 |
|  |  |  |  |
| **Delivery room resuscitation** |  |  |  |
| Delayed cord clamping, n (%) | 200 (51.3) | 67 (60.9) | 0.074 |
| No, n (%) | 190 (48.7) | 43 (39.5) | **0.027** |
| 30-60 seconds, n (%) | 160 (41.0) | 45 (41.3) |  |
| 61-120 seconds, n (%) | 40 (10.3) | 21 (19.3) |  |
| Cord milking, n (%) | 24 (6.2) | ^a^ | 0.936 |
| Nasal continuous positive airway pressure, n (%) | 275 (70.5) | 83 (75.5) | 0.310 |
| Positive pressure ventilation via mask and / or noninvasive ventilation, n (%) | 306 (78.5) | 91 (82.7) | 0.329 |
| Endotracheal tube ventilation, n (%) | 172 (44.1) | 48 (43.6) | 0.931 |
| Chest compression and / or epinephrine, n (%) | ^a^ | ^a^ | 0.464 |
| 5-minute FiO2, median (interquartile range) | 50.0 (30.0-91.0) | 40.0 (30.0-80.0) | **0.008** |
| 21-30%, n (%) | 102 (26.2) | 44 (40.0) | **0.019** |
| 31-60%, n (%) | 134 (34.5) | 32 (29.1) |  |
| 61-100%, n (%) | 153 (39.3) | 34 (30.9) |  |
| 1-minute Apgar score, median (interquartile range) | 4.0 (2.0-6.0) | 5.0 (2.0-7.0) | 0.487 |
| 0-3, n (%) | 150 (38.6) | 38 (34.6) | 0.587 |
| 4-6, n (%) | 150 (38.6) | 42 (38.2) |  |
| 7-10, n (%) | 89 (22.9) | 30 (27.3) |  |
| 5-minute Apgar score, median (interquartile range) | 7.0 (6.0-8.0) | 8.0 (7.0-8.0) | 0.060 |
| 0-3, n (%) | 39 (10.0) | ^a^ | **0.026** |
| 4-6, n (%) | 97 (24.9) | 15 (13.6) |  |
| 7-10, n (%) | 254 (65.1) | 86 (78.2) |  |

^a^ Cell counts <12 were suppressed in order to protect subject privacy. Data may not add up to 100% due to missing data.


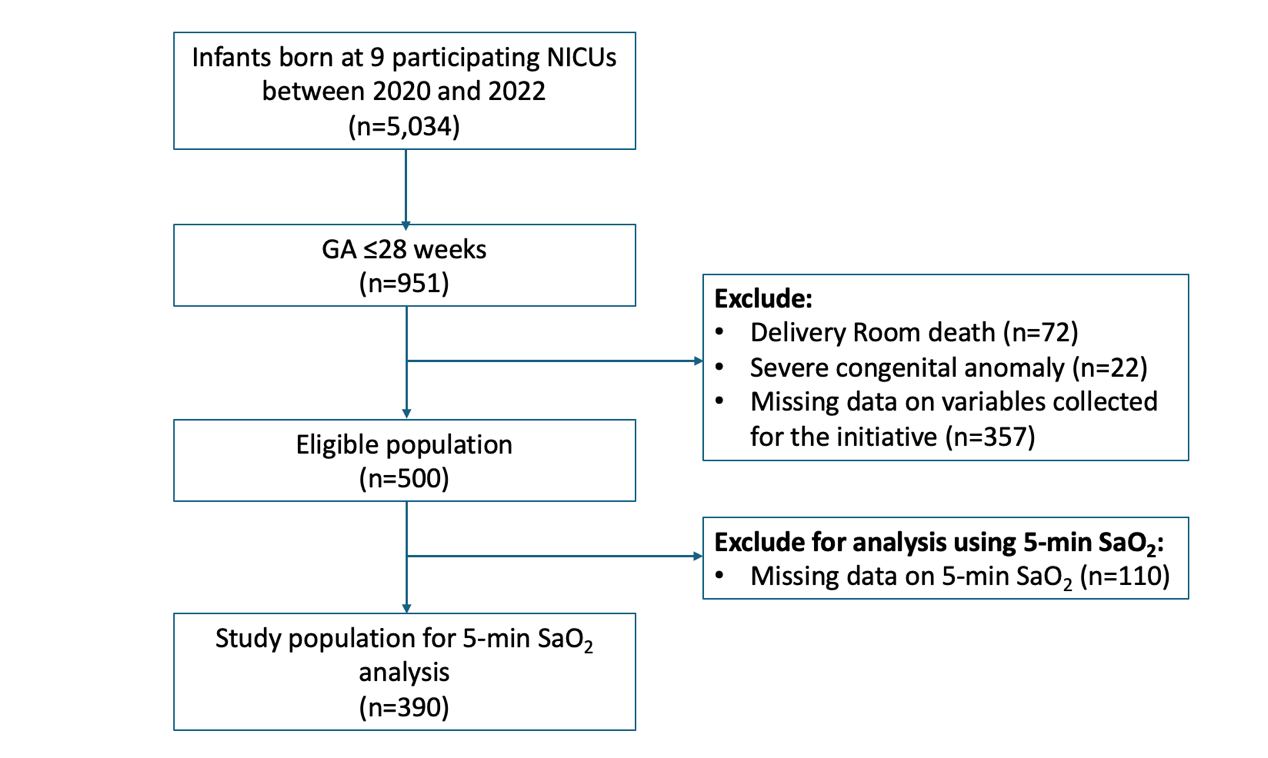


**Supplemental Figure 1. Study Population**

**Supplemental Figure 2. Distribution of 5-min SpO2 among extremely preterm infants in participating sites**


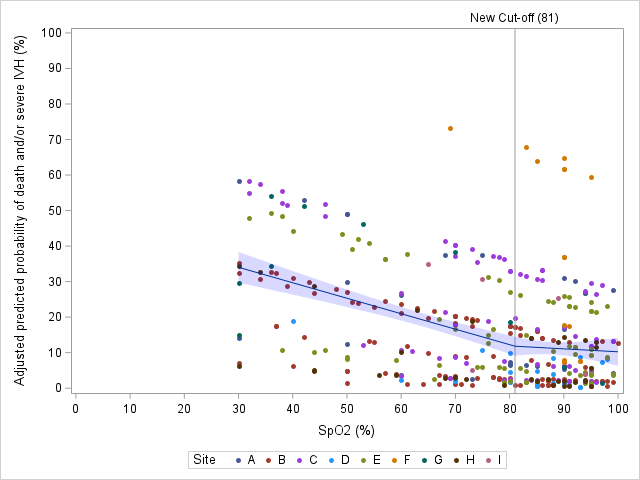


**Supplemental Figure 3. Adjusted predicted probability of death and / or severe IVH and 5-minute SpO2 by excluding records with 5-minute SpO2 less than 30%:** An optimal breakpoint was identified at a 5-minute SpO2 of 81%. Below this 81% threshold, the adjusted probability of death and / or severe IVH significantly decreased with increasing 5-minute SpO2 (slope -4.6%, 95% CI (-6.0%, -3.3%), P-value < 0.001); while above 81%, the adjusted probability of death and / or severe IVH was not significantly associated with 5-minute SpO2 (slope -0.8%, 95% CI (-2.6%, 1.0%), P-value = 0.369). Scatter plot: Predicted probability of death and / or severe IVH by 5-minute SpO2; Fitted lines with 95% confidence intervals


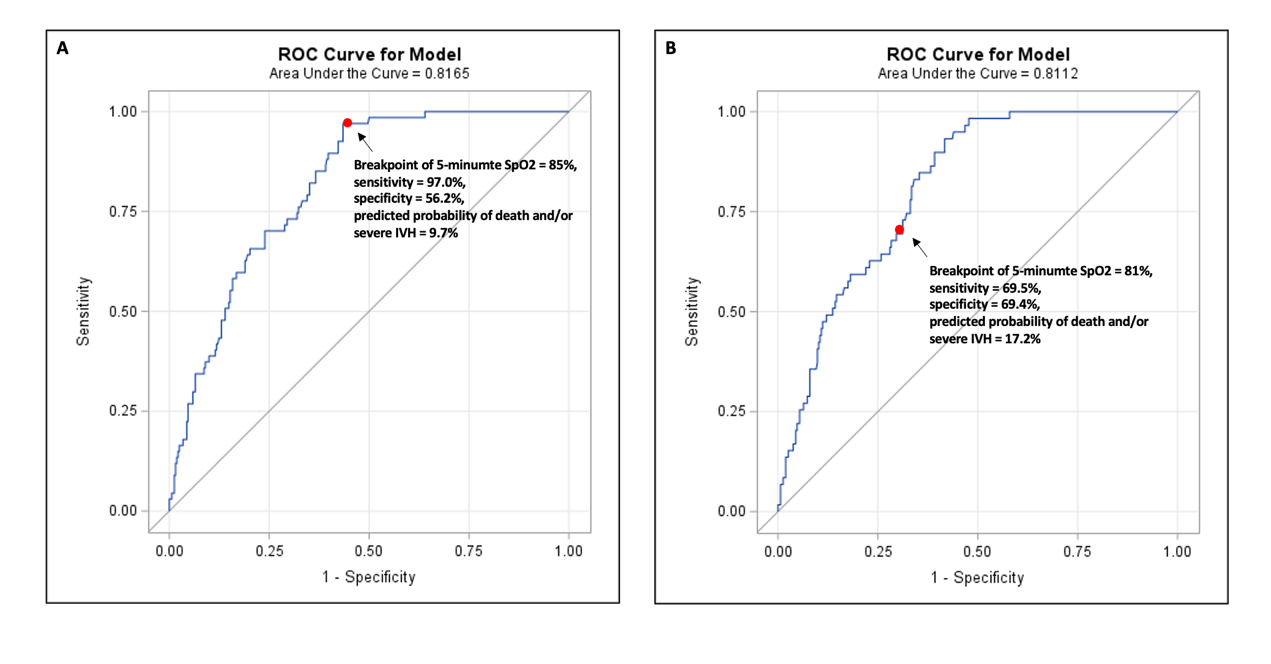
 **Supplementary Figure 4. ROC curve and optimal breakpoint identified from the ROC analysis**

A: At optimal breakpoint of 5-minute SpO2 = 85%, sensitivity = 97.0%, specificity = 56.2%, the predicted probability of death and / or severe IVH was 9.7%.

B: By excluding records with 5-minute SpO2 less than 30%, at optimal breakpoint of 5-minute SpO2 = 81%, sensitivity = 69.5%, specificity = 69.4%, the predicted probability of death and / or severe IVH was 17.2%.
